# Short-term results of the combined application of neodymium-doped yttrium aluminum garnet (Nd:YAG) laser and erbium-doped yttrium aluminum garnet (Er:YAG) laser in the treatment of periodontal disease: a randomized controlled trial

**DOI:** 10.1007/s00784-021-03911-x

**Published:** 2021-04-04

**Authors:** Markus Laky, Maximilian Müller, Brenda Laky, Muazzez Arslan, Christian Wehner, Selma Husejnagic, Stefan Lettner, Andreas Moritz, Xiaohui Rausch-Fan

**Affiliations:** 1 Division of Conservative Dentistry and Periodontology, Medical University of Vienna, Sensengasse 2a, 1090 Vienna, Austria; 2 Karl Donath Laboratory for Hard Tissue and Biomaterial Research, Medical University of Vienna, Sensengasse 2a, Vienna, Austria; 3 Austrian Cluster for Tissue Regeneration, Vienna, Austria; 4 Division for Dental Student Training and Patient Care, Medical University of Vienna, Sensengasse 2a, 1090 Vienna, Austria

**Keywords:** Erbium laser, Laser therapy, Neodymium laser, Non-surgical treatment, Periodontal debridement, Periodontal disease, Solid-state lasers

## Abstract

**Objectives:**

Nd:YAG and Er:YAG lasers have been previously used as an adjunct in periodontal therapy. The aim of this single-blinded randomized controlled clinical trial was to evaluate the efficacy of a combined application of Nd:YAG and Er:YAG laser irradiation in periodontal treatment.

**Materials and methods:**

Twenty-two patients with at least one site of ≥ 6 mm periodontal probing depth (PPD) after mechanical debridement with curettes and sonic instruments at periodontal reevaluation were included in the study. Patients were randomly allocated at a 1:1 ratio to either a combined Nd:YAG/Er:YAG laser therapy (test group) or a “turned off” laser therapy (control group). The Nd:YAG laser was used for periodontal pocket deepithelialization and to stabilize the resulting blood clot. The Er:YAG laser was primarily used for root surface modification. PPD (mm), clinical attachment level (CAL, mm), and bleeding on probing (BOP, +/−) at the site of laser treatment were evaluated at baseline and 2 months after treatment.

**Results:**

The mean improvements from baseline to 2-month follow-up for PPD were significantly better in the laser group (2.05 ± 0.82 mm) compared to the control group (0.64 ± 0.90 mm; *p* = 0.001). Likewise, the gain in CAL was significantly better in the laser group (1.50 ± 1.10 mm) than in the control group (0.55 ± 1.01mm; *p* = 0.046).

**Conclusions:**

The combined application of Nd:YAG and Er:YAG laser irradiation as an adjunct to conventional non-surgical therapy showed a significant beneficial effect on periodontal treatment results.

**Clinical relevance:**

Combined Nd:YAG and Er:YAG laser irradiation could be a useful procedure additionally to conventional non-surgical periodontal therapy to improve periodontal treatment results.

**Clinical trial registration:**

ISRCTN registry #ISRCTN32132076

## Introduction

Periodontitis is one of the most common diseases worldwide and prevalence increases gradually with age showing a sharp increase between the third and fourth decade in life [1]. The initial part of periodontal treatment consists of a reduction of known risk factors (e.g., smoking) and subgingival debridement as a minimal invasive procedure, aiming to eliminate bacterial biofilm from the root surface. However, treatment outcomes may not always be sufficient for moderate to deep pockets in terms of periodontal probing depth reduction [2]. Thus, at sites with remaining high probing depths after initial therapy, surgical procedures may be performed in order to reduce periodontal probing depths and to achieve regeneration of lost periodontal tissues [3, 4]. However, predictable treatment of deep periodontal pockets without surgery or with minimally invasive periodontal surgical procedures would be beneficial for many patients with severe periodontal disease [2].

In recent years, lasers have been increasingly investigated as an adjunctive treatment method in periodontal therapy [5]. The Er:YAG laser has a good capability of ablating hard tissues like dentin or dental calculus as well as soft tissue [6–9]. Erbium lasers are used in endodontic treatment not only for smear layer removal and activation of rinsing solutions [10], but they also exhibit a strong antibacterial effect [11]. Erbium lasers are also considered as appropriate devices for root surface modifications. Fibroblast attachment on Er:YAG laser-treated surfaces is significantly increased compared to scaling and root planing [12]. Hence, erbium lasers might be suitable tools in periodontal therapy [2].

The Nd:YAG laser emits a light with a wavelength of 1064 nm and is readily and selectively absorbed in areas of inflammation by blood components and pigmented tissue. The Nd:YAG laser light is generally used in periodontal treatment for bacterial inactivation, removal of periodontal pocket epithelium, and hemostasis in inflamed tissue [13]. Additionally Nd:YAG laser irradiation was shown to inactivate *Candida albicans* [14].

As the erbium laser light might be especially advantageous on the hard tissue root surface and the Nd:YAG laser has additional positive effects on hemostasis and bacterial reduction, a combined treatment with both lasers could be beneficial in periodontal therapy. The main aim of the present randomized controlled trial was to evaluate short-term effects of an adjunctive combined Er:YAG and Nd:YAG laser irradiation after conventional mechanical debridement at sites with PPD ≥ 6 mm in patients with periodontal disease.

## Methods

### Study design

For this prospective, 1:1 randomized, single-blinded (patient), controlled clinical trial, patients were consecutively recruited from January 2018 to December 2018 at the Division of Conservative Dentistry and Periodontology and at the Division for Dental Student Training and Patient Care, Medical University of Vienna, Austria. The study was approved by the human subjects ethics board of the Medical University of Vienna (1747/2017) and was conducted in accordance with the Helsinki Declaration of 1964, as revised in 2013. All participating patients signed informed consent prior to commencement of the study.

**Fig. 1:**
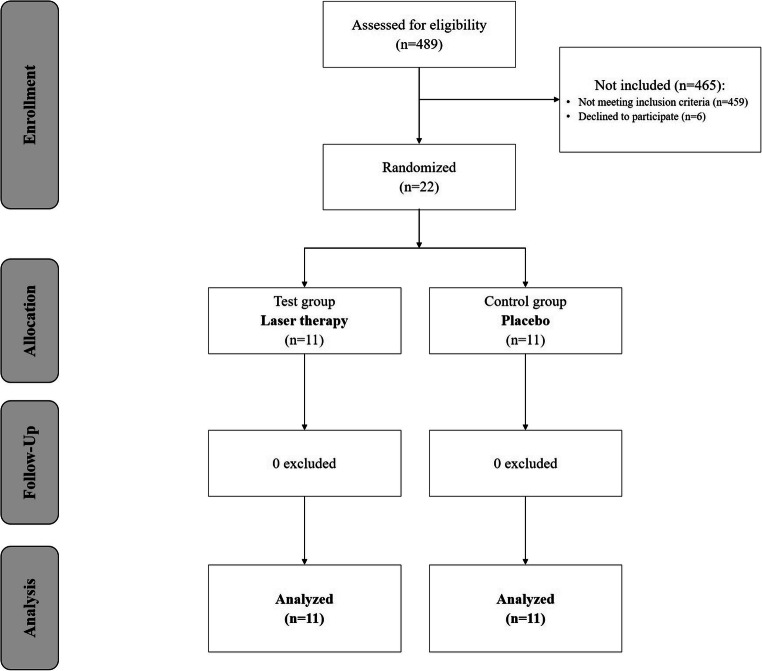
Consolidated Standards of Reporting Trials (CONSORT) flow chart of participants

Four hundred eighty-nine patients (Fig. 1), who received periodontal treatment at the Division of Conservative Dentistry and Periodontology and at the Division for Dental Student Training and Patient Care, were screened for eligibility with the following inclusion criteria: at least one site with a probing depth of ≥ 6 mm about 8 weeks post-completion of initial supra- and subgingival periodontal debridement (scaling and root planing) at periodontal reevaluation. Assessment of PPD and CAL was performed at six sites per tooth. The exclusion criteria were systemic diseases, which could potentially influence the outcome of the therapy (e.g., diabetes mellitus, pregnancy, immunosuppression, malignant diseases) and furcation involvement of the tooth with laser irradiation.

Twenty-two systemically healthy patients were enrolled as per protocol and randomly assigned to a test group (laser on, *n* = 11) and a control group (laser off, *n* = 11). The 1:1 randomization was performed by flipping a coin. The patients did not know whether they were in the test group or in the control group. The laser treatment in the control group was performed with a switched off laser device. PPD and CAL changes as well as BOP were assessed 8 weeks after treatment at the site with laser irradiation/sham irradiation.

### Laser treatment

A local anesthetic lidocain-gel (Xylocain 2% Gel, AstraZeneca, Wedel, Germany) was applied 2 min before the laser treatment. After periodontal probing with a CP12 periodontal probe (Hu-Friedy, Frankfurt, Germany) subgingival debridement was performed with a sonicflex (KAVO, Biberach, Germany) instrument tip #60. According to the TwinLight^TM^ protocol in the test group, removal of the pocket epithelial lining was done with the Nd:YAG laser LightWalker (Fotona, Ljubljana, Slovenia) for 30 s at the treatment site without any water cooling in contact mode. Laser settings were micro-short pulse (pulse duration 100 μs), 2.5 W, 125 mJ, 20 Hz, 300 μm, fluence 176.8 J/cm^2^ with an initiated fiber without water cooling. As the wavelength and fiber of the Nd:YAG laser is comparable to diode laser irradiation in our opinion, an initiated tip has advantages in pocket deepithelialization [15]. Then a 60-s irradiation of the Er:YAG laser LightWalker was applied on the root surface with water cooling with a flow rate of 44 ml/min in contact mode. The laser settings were micro-short pulse (pulse duration 100 μs), 40 mJ, 40 Hz, with a Varian 400 μm tip, fluence 31.8 J/cm^2^. The tip was inclined 10–15° to the root surface. Finally, 30-s stabilization of the forming blood clot was accomplished with a Nd:YAG laser (very long pulse, pulse duration 600 μs, 3.5 W, 175 mJ, 20 Hz, 300 μm fiber, fluence 247.6 J/cm^2^). In the control group, all the steps were performed without activating the laser device. Laser treatment was performed by ML. Re-assessment was performed 8 weeks post-treatment by senior periodontal staff members of the Division of Conservative Dentistry and Periodontology (ML, CW).

### Clinical assessment

Data were collected before and at least 2 months after laser therapy. Demographic parameters included age (years), gender (female/male), involved tooth, and site. The primary outcome was PPD (mm) and the secondary outcome variables included CAL (mm) and BOP (+/−) at the site of laser irradiation.

### Statistical analyses

A priori sample size calculation revealed that 20 patients (10 patients in each group) would provide a power of 85% with a two-sided 0.05 alpha level to detect significant differences in PPD, assuming a mean difference of 0.7 mm and a standard deviation of 0.5 mm. Assumptions were based on a previous publication by Crespi et al. [16]. To compensate for an eventual loss of recruited patients during follow-up, we increased the number of patients to a total of 22 (11 patients per group).

Data distribution was assessed by visual inspection of histograms and the Kolmogorov-Smirnov test. Normally distributed continuous data were presented as means with standard deviation, otherwise as median and range. Categorical variables were described as proportions and frequency counts. Independent *t*-tests or the non-parametric Mann-Whitney *U* tests were used to compare continuous variables between the study and control group. Paired *t*-tests or non-parametric Wilcoxon signed-rank tests were used to compare scores between two time points. Categorical data were assessed using Fisher’s exact or chi-square tests between the study and the control group. McNemar was used to compare categorical data between two time points. All data were analyzed using commercially available software (Excel, Microsoft Office 2013; SPSS v23; IBM, Chicago, IL, USA). Statistical significance was set at the conventional *p* value of <0.05 (two-sided).

## Results

Despite randomization, the mean age in the control group (49.0±9.1) was significantly lower than the mean age in the test group (61.1±10.7). No other significant differences in patient characteristics were detected between the test (laser on) and the control (laser off) group (Table 1).

**Table 1:** Patient baseline characteristics

	Test group Laser on (*n*=11)	Control group Laser off (*n*=11)	*p* value
Age, mean ± SD, y	61.1±10.7	49.0±9.1	0.010^a^
Gender, female/male, *n* (%)	2 (18.2%)/9 (81.8%)	4 (36.4%)/7 (63.6%)	0.635^b^
Involved tooth			
Incisors Canine Premolar Molar	1 (9.1%) 2 (18.1%) 4 (36.4%) 4 (36.4%)	2 (18.2%) 0 1 (9.1%) 8 (72.7%)	0.141^b^
Involved site			
Disto-buccal Disto-palatinal/lingual Mesio-buccal Mesio-palatinal/lingual	5 (45.4%) 3 (27.3%) 2 (18.2%) 1 (9.1%)	4 (36.4%) 5 (45.4%) 1 (9.1%) 1 (9.1%)	0.567^b^

*Abbreviations*: *BMI*, body mass index; *SD*, standard deviation

^a^Independent *t*-test

^b^Chi-square test

The mean follow-up time for all patients was 2.5±1.6 months. No complications/adverse events were detected.

Comparisons of all parameters are presented in Table 2. Significant improvements from baseline to the short-term (2 months) follow-up were found regarding PPD in both groups and CAL in the laser group. While in the laser group BOP decreased in five patients (45.5%), no change in BOP was detected in the control group.

**Table 2:** Outcome parameters

	Test group Laser on (*n*=11)	Control group Laser off (*n*=11)
PPD (mm)		
Baseline	6 (6–9)	6 (6–9)
2 months	5 (3–6)	6 (4–7)
*p*-value (within group)	0.003^a^	0.038^a^
CAL (mm)		
Baseline	8 (6–11)	7 (6–14)
2 months	6 (5–10.5)	7 (5–12)
*p*-value (within group)	0.005^a^	0.111^a^
BOP (+/−)		
Baseline	9/2	7/4
2 months	4/7	7/4
*p*-value (within group)	0.063^b^	0.999^b^
API (%)		
Baseline	20.5±17.3	12.6±11.5
2 months	19.3±17.4	20.8±23.4
*p*-value (within group)	0.800^c^	0.137^c^

For PPD and CAL, the median and the lower and upper range are shown

*Abbreviations*: *API*, approximal plaque index; *BOP*, bleeding on probing; *CAL*, clinical attachment level; *PPD*, periodontal probing depth

^a^Wilcoxon signed-rank tests

^b^McNemar test

^c^Paired *t* test

**Fig. 2:**
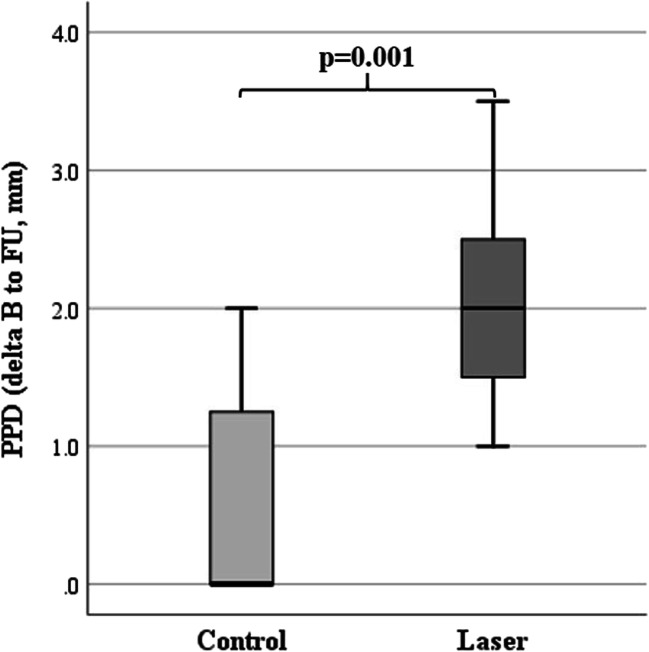
Box plot showing significantly better periodontal probing depth (PPD, mm) differences between baseline (B) and at 8-week follow-up (FU) of the laser (*n*=11) compared to the control (*n*=11) group

PPD differences from baseline to follow-up were significantly better in the laser (2.05 ± 0.82 mm) compared to the control group (0.64 ± 0.90 mm; *p*=0.001; Fig. 2).

**Fig. 3:**
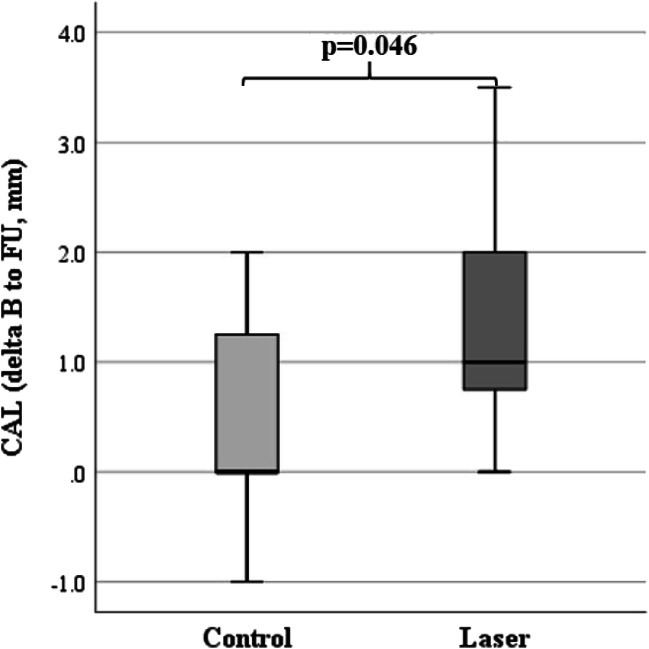
Box plot showing significantly better clinical attachment levels (CAL, mm) differences between baseline (B) and at 8-week follow-up (FU) of the laser (*n*=11) compared to the control (*n*=11) group

CAL differences from baseline to follow-up were significantly better in the laser (1.50 ± 1.10 mm) than in the control group (0.55 ± 1.01 mm; *p*=0.046; Fig. 3).

After treatment, there were more patients with PPD ≤ 4 mm in the laser group (27.3 %) than in the control group (18.2 %).

## Discussion

The results of our combined Nd:YAG/Er:YAG laser intervention showed a significant reduction in PPD and a significant gain in CAL at the sites of laser treatment compared to the control group. To the best of our knowledge, this is the first time the TwinLight^TM^ laser treatment was investigated at periodontal pockets ≥ 6 mm additional to scaling and root planing. The Nd:YAG/Er:YAG laser treatment is a minimally invasive intervention. However, it is possible to perform crucial elements of periodontal regenerative surgical procedures like removal of the pocket epithelium, root surface conditioning, and stabilization of a blood clot with the lasers and without raising a full periodontal flap. It was demonstrated that the application of both the Nd:YAG laser and the Er:YAG laser can achieve an ablation of the epithelial lining of the periodontal pocket in a minimally invasive way [17, 18]. Laser-assisted new attachment procedures (LANAP^®^) with removal of epithelial structures and stabilization of a blood clot with a Nd:YAG laser have been described in the literature, and there is human histologic evidence for cementum-mediated periodontal ligament attachment in the absence of a long junctional epithelium [2, 18–20]. We consider the removal of epithelial structures an important step for periodontal regeneration and sustained periodontal pocket closure. Additionally, the Er:YAG laser in the TwinLight^TM^ protocol is used in a second step to create an optimal root surface for periodontal regeneration. Er:YAG laser irradiation can be used not only for calculus removal [21] from the dental root surfaces but also for root surface conditioning. In vitro studies have revealed an increased attachment of human periodontal ligament fibroblasts on Er:YAG laser-irradiated root surfaces [12, 22, 23]. Erbium-laser irradiation may result in an ameliorated dental root surface for fibroblast cell attachment and is possibly an important factor for improved periodontal healing.

As a final treatment procedure in the TwinLight^TM^ laser application protocol, the Nd:YAG laser aids in sustaining and stabilizing a blood clot in the periodontal pocket at the site of the intervention [18]. Removal of pocket epithelium, root surface modification, and blood clot stabilization which are likely to be performed in regenerative periodontal surgical interventions may also be achieved by means of the minimally invasive treatment with the combination of the two laser lights, thus avoiding a surgical incision.

The application of the Nd:YAG laser on dental root surfaces has always been challenging in periodontal treatment, since subgingival calculus often has a dark color. Thus, side effects such as thermal damages to the root surfaces may occur due to high laser light absorption in pigmented calculus deposits on the dental root surface [24]. An additional benefit to the Nd:YAG treatment procedures seems to be its combination with the hard tissue ablation effects of the Er:YAG laser on the root surface. The Nd:YAG laser is used for reshaping oral soft tissues, for inactivating bacteria, and for achieving hemostasis in the periodontal pocket through heat-induced coagulation [2, 25]. The Nd:YAG laser has a strong bactericidal effect on the most common putative periodontal pathogens [26]. Most nonsporulating bacteria, including periodontopathic anaerobes, are readily deactivated at 50°C [13].

Saglam et al. [27] found a significant improvement in PPD and CAL in the combined Nd:YAG/Er:YAG application in a split-mouth design compared to scaling and root planing alone in periodontal pockets ≥ 7 mm. However, in the respective study, no conventional scaling and root planing were performed in the laser-treated areas. In our study protocol, scaling and root planing with hand instruments and sonic instruments were performed before the laser treatment, and we focused on applying our intervention only at sites with periodontal probing depths ≥ 6 mm after initial therapy at periodontal reevaluation. Grzech-Leśniak et al. [28] reported that the combination of Nd:YAG and Er:YAG lasers significantly improved the microbiological and clinical outcomes of non-surgical periodontal therapy. Sanz-Sánchez et al. [29] reported a significant lower percentage of sites with PPD ≥ 4.5 mm in the group with additional Er:YAG laser treatment compared to full mouth scaling and root planing up to 1 year. The laser treatment was performed 1 week after conventional full mouth debridement at sites ≥ 4.5 mm.

In a systematic review on the application of the Er:YAG laser in periodontal therapy, Zhao et al. [30] found no advantage when the laser was used as an alternative treatment to scaling and root planing without conventional debridement, but the group reported a significant difference when the erbium laser was used as an adjunct to scaling and root planing. Conventional debridement is the basis of periodontal therapy. We suggest that the laser should be used additionally to this basic conventional treatment. Regular sonic/ultrasonic devices and hand instruments are suitable tools to perform cleaning procedures for reducing biofilm. It is not cost and time efficient to replace those simple and long-lasting cleaning devices with an expensive and, in the medium-term, error-prone laser device. In periodontal treatment, the laser tip in the periodontal pocket is very close to the laser target tissues. This results in a reduced lifetime of the laser tip due to the debris produced by the laser ablation, which to a certain extent may damage the laser tip. Therefore, we recommend an adjuvant treatment protocol using the TwinLight^TM^ laser to be applied only on those sites with deep PPD remaining after regular scaling and root planing instead of using the laser in initial periodontal treatment on all root surfaces on each tooth.

The application of the Nd:YAG laser and the Er:YAG laser offers the combined advantages of both the Nd:YAG laser, such as bacterial reduction and clot stabilization, and the good hard tissue ablating and surface-conditioning properties of the Er:YAG laser. The provision of both laser wavelengths for dental treatment requires a certain amount of financial investment for the acquisition and maintenance of the laser devices, and a thorough economic calculation is definitely recommended before the purchase of any laser. Our results showed that the combined use had a significant effect on periodontal treatment outcomes. An improvement in PPD was also observed in the control group after additional conventional scaling and root planing. Thus, the additional cleaning and the participation in the study with a probably increased awareness of the periodontal condition resulted in reduced PPD.

Limitations of this study were a short 2-month follow-up after the laser intervention and a missing bacterial evaluation. Despite randomization, more molar teeth were found in the control group than in the test group. This could have had an impact on the treatment results; however, as we excluded an existing furcation involvement in molar teeth, we consider it less critical for the reported results. Another limitation of the study, despite randomization, was the age difference between the test group and the approximately 10-year younger control group. The progression of periodontitis might have been more slowly in the older test group, and this could have had an effect on the response to the laser treatment. However, generally one would expect the younger control group to respond in a better way to regenerative procedures. Hence, we believe that the age difference between the groups does not jeopardize the validity of our results.

## Conclusions

In conclusion, the combined application of Nd:YAG and Er:YAG laser irradiation as an adjunct to conventional non-surgical therapy showed a significant beneficial effect on periodontal treatment results.
